# The Prognosis and Treatment of Ankle-Joint Disease

**Published:** 1882-05

**Authors:** 


### ORIGINAL TRANSLATIONS AND ABSTRACTS.

### THE PROGNOSIS AND TREATMENT OF ANKLE-JOINT DISEASE.

Newton M. Shaffer, M. D. (*Annals of Anatomy and Surgery*, May, 1882). In this article he calls attention to two important conditions of the ankle-joint, viz., chronic synovitis and chronic osteitis; but for convenience he classes under the last term a variety of pathological conditions, as (1) *osteitis fungosa* or *granulosa* (primary when occurring in epiphyseal structures, or secondary as a sequence of fungous degeneration of the synovial tissue); (2) *caries necrotica* (in which the process of destruction is quite rapid and carious bone is discharged); (3) *simple superficial caries* (following periostitis); and (4) *caries interna caseosa.* The prognosis varies somewhat in all these as well as the treatment. One element of the prognosis is time; and an unfavorable prognosis should always be the rule when the initial symptoms are apparently unimportant and the onset insidious. But in childhood, unless the case is a very bad one, a good result may be expected, especially in ankle-joint disease. The prognosis, however, should not be exact, but limited rather to a description of the nature of the treatment and the care necessary to obtain a good result. Caries fungosa is a very sluggish condition, with very insidious prodromata. It is accompanied sometimes by an atonic form of suppuration; the pus is not laudable, but profuse, thin and ichorous and filled with broken-down tissue. In the necrotic caries the prognosis is more favorable ; nature rapidly exfoliates the diseased bone ; but in these cases there is more marked constitutional disturbance. The process more nearly approaches the acute form of disease, its course is shorter, recovery with anchylosis taking place in many instances.

The superficial caries, if it does not involve the articular surfaces, may be called a favorable lesion if properly recognized and treated. It may, however, become the caries necrotica and total joint destruction may ensue. A favorable type of suppuration frequently accompanies this process, and the rule should be to open as early as possible. The caries interna caseosa is a form very difficult to recognize in the early stage, and is very apt, with caries fungosa, to be called tuberculosis of the joint.

It is not possible to clinically differentiate between these two conditions. In chronic synovitis we find the joint motions nearly normal ; a bulging of the joint capsule, an elastic feeling very suggestive of fluctuation, a well-rounded calf matching its fellow, and an unimpaired walk and attitude. In chronic osteitis the joint motions are very materially impaired by a very pronounced reflex muscular spasm, simulating anchylosis, the joint outline is not obliterated, and the calf is smaller compared with its mate.

The secret of successfully treating chronic joint-disease lies in its early recognition. Teach mothers and nurses that many limps mean something serious ; that many cases of "worms" and "indigestion" turn out to be humpback. In simple hydrarthrosis, the absorbents still act, and our greatest hope in the treatment of chronic synovitis is that the free surface of the membrane is not disintegrated by fungous masses. Upon this basis we make use of compression, by the use of an elastic silk anklet, compressed sponge, or a firmly applied flannel bandage frequently readjusted. To excite the absorbents to greater action we use oleate of mercury, iodine, blisters, friction by the hands, and the actual cautery. If the joint shows a higher temperature than normal avoid excitants and apply ice and cooling applications as long as necessary. Do not confine yourself long to any one internal remedy ; use iron, the various iodides, mercury, hypophosphites, etc., according to indications ; but iron is always indicated, and the favorite prescription of Shaffer is plain tincture of the chloride in large doses, combined with an equal quantity of glycerine ; it seems most to aid the plastic power. There is no remedy which acts specifically on fungoid degeneration ; therefore we are compelled to use thorough systemic treatment. Traumatism may be an uncertain element in the causation of chronic joint disease, but no one doubts its relation to the progress of the lesion when once established, when once a joint or any of its essential structures become diseased. It is important in a case of diseased joint that we should adopt means which will effectually remove the element of constant traumatism produced by locomotion, while at the same time locomotion and exercise should not be materially interfered with. While the patient is sitting there need be nothing but protection against accidental traumatism, but when he walks this protection must be absolute, and *the means of protection should act promptly and without the exercise of volition on the part of the patient.* The vulnerable point must always be protected both against the natural use of the part and from the patient's own indiscretion and accident. Partial relief does harm, because the patient, feeling the relief, gives the joint more liberty, and relapse at once ensues. Devise and prescribe the instrument yourself, and see that the principle involved is carried out in its construction ; it is as irrational to go to the instrument-maker for advice as to send a patient to a pharmacist for treatment.

In *chronic synovitis* do not immobilize the joint, or it will soon become stiff and useless. Motion without pressure is demanded. To accomplish this we need a certain amount of traction, but we must not prevent free movement. Clinical experience teaches, in ankle-joint synovitis, that, if we apply an apparatus that just prevents the foot from touching the floor when the patient walks, we place the joint in the best possible local condition for repair. Shaffer has been disappointed with all instruments devised for the treatment of ankle-joint disease, whether synovial or osseous, that act on the ankle-joint alone. To perfectly protect the ankle-joint we must limit more or less the movements of the knee-joint ; if we wish to immobilize the ankle-joint in locomotion we must also control the knee. We must go to the perineum for our fixed point, to the tuber ischii, just as we do in hip disease ; we must not depend on adhesive plaster and to its attachment to the skin for our counter-support —we cannot make a reliable fixed point with adhesive plaster.

The author's instrument is modified from one introduced by Dr. Taylor for chronic synovitis of the knee-joint ; it has a peculiar arrangement by which the patient can flex the knee-joint and the hip-joint as he sits down, and this mechanism locks automatically in the straight position when the patient stands or walks. It is composed of six essential parts. (1) The pelvic band, which enables us to make the supplementary perineal bands fixed points of support ; (2) an automatic hipjoint movement, which admits of flexion when the patient sits down, and which locks without aid when he stands ; (3) the thigh-pait, containing an extension rod, to which is attached a lateral plate for loose lateral attachment to the thigh ; (4) the knee-joint, with its automatic "snap," which always locks when the patient stands up and commences to walk ; (5) the calf-part, having a band-and-leather lacing to afford lateral attachment ; (6) the ankle-joint, with a worm and screw controlled by a key, enabling us to place the foot-piece in any desirable antero-posterior position. The joints at the knee and ankle permit the patient to sit in an easy position, and when the patient stands they each lock automatically and form a continuous splint ; traction is always present when the patient walks or stands, and protection, with traction if desired, is thus always secured when the patient sits.

In chronic synovitis we limit flexion and extension of the foot to any desired extent by a "catch" in the side-piece of the foot-plate. The instrument is generally applied without the aid of adhesive plaster ; we adjust the foot-plate closely in contact with the sole of the foot before the shoe is applied ; then lace the calf piece and apply the shoe over the foot-piece. Now lace the shoe singly ; lace the thigh-piece ; buckle the pelvic band and secure the perineal pads. Now make gentle traction with the key just enough to carry the foot-piece (enclosed by the shoe) slightly away from the sole of the foot. By removing a couple of screws the automatic spring can be taken off, and the knee-joint is rendered stiff if you wish. The patient should never walk without this support, and it gives so much comfort that he prefers to wear it : it can be worn even at night if you wish, by applying a bandage in place of the shoe.

In chronic osteitis of the ankle the instrument must be modified to meet the different pathological state. Adapt the apparatus *exactly* to the deformity, and by gradual and very gentle changes restore the joint to the normal position. By means of the antero-posterior worm and screw we can, with the key, turn the foot into a position of equinus or calcaneus, and so adapt it to the deformity ; there is no lateral malposition of the joint itself and we can bend the side-bar to meet the secondary tarsal malpositions. After adjusting the foot-piece to the actual deformity, the process of adjustment is the same as before ; in addition we can reinforce the laced shoe with adhesive plaster, making very thorough traction. It is only necessary to make the sound limb artificially longer with a high-soled shoe and advise care in locomotion at first. These patients do not need crutches or cane ; they become very active.

By gradually changing the position of the foot-plate, the foot follows it, and in a few days the ankle-joint is in a good position. The presence of abscesses does not interfere with the use of this support. If pain is severe, apply adhesive plaster and keep the patient in bed, where proper local treatment can be carried out. He has seen but few cases of ankle-joint disease where excision or amputation is justifiable in a child.

